# Some Prescriptions Discussed—I

**Published:** 1909-05-08

**Authors:** 


### THERAPEUTICS AND PHARMACY.

### SOME PRESCRIPTIONS DISCUSSED—I.

"A SCOTTISH PHARMACIST" some time ago dispensed personally a number of the formularies recommended in the British Pharmaceutical Codex, and in local pharmacopœias, such as that in use in Glasgow and the West of Scotland, and he gives the results he has found in some interesting papers in the *Pharmaceutical Journal.* Sometimes the prescriptions yielded medicines that looked well and remained in good condition for a long time; sometimes, on the other hand, the results were not so good; and in view of the efforts that are now being made to revert to personal prescription-writing, rather than to order proprietary medicines by number, or in other similar ways, it is a matter of some interest to note which prescriptions are satisfactory from the point of view of dispensing, and which are not. In the original papers the quantities are written as percentages, each formulary being made up to one hundred parts. To make the prescription up to an ounce instead of to one hundred parts, it is approximately correct to multiply the quantity of each ingredient by five to give the amounts required of each, in grains or minims. If absolute accuracy is required, it would be necessary to make the mixtures up to 500 instead of to 480 minims.

The following was found to be a very excellent way of prescribing cod-liver oil:

EMULSIO OLEI MORRHUÆ COMPOSITA, B.P.C.

| | Parts. |
|---|---|
| Cod-liver oil ... ... ... ... | 50.00 |
| Yolk of egg (by volume) ... ... ... | 6.50 |
| Tragacanth (in powder) ... ... ... | 0.25 |
| Elixir of gluside ... ... ... ... | 0.75 |
| Simple tincture of benzoin ... ... | 0.75 |
| Spirit of chloroform ... ... ... | 3.00 |
| Essential oil of bitter almonds ... ... | 0.10 |
| Distilled water, sufficient to produce ... | 100.00 |

It is often desired to give olive oil in quantity to patients, for instance in cases of gall-stones, and a precisely similar prescription for olive oil emulsion yields an excellent product, entitled Emulsio Olei Olivæ Composita.

When it is desired to prescribe hypophosphites along with cod-liver oil, as in more than one well-known proprietary preparation, the formulary recommended in the codex, is the following:—

EMULSIO OLEI MORRHUÆ CUM HYPOPHOSPHITIBUS, B.P.C.

| | Parts. |
|---|---|
| Cod-liver oil ... ... ... ... | 50.00 |
| Yolk of egg (by volume) ... ... ... | 6.50 |
| Tragacanth (in powder) ... ... ... | 0.25 |
| Elixir of gluside ... ... ... ... | 0.75 |
| Simple tincture of benzoin ... ... | 0.75 |
| Spirit of chloroform ... ... ... | 3.00 |
| Oil of bitter almonds ... ... ... | 0.10 |
| Sodium hypophosphite ... ... ... | 0.75 |
| Calcium hypophosphite ... ... ... | 0.75 |
| Distilled water ... ... ... ... | 100.00 |

It has been found, however, that whereas this prescription gives a good product when it is newly dispensed, in a week or two it becomes too thick to pour from a narrow-mouthed bottle. It is more satisfactory when only nine-tenths of the above quantity of tragacanth is employed.

The prescription which is equivalent to Parrish's syrup or chemical food, is the following:—

SYRUPUS FERRI PHOSPHATIS COMPOSITUS, B.P.C.

| | Parts. |
|---|---|
| Iron wine ... ... ... ... ... | 0.40 |
| Concentrated phosphoric acid ... ... | 7.50 |
| Precipitated calcium carbonate ... | 1.35 |
| Potassium bicarbonate ... ... ... | 0.01 |
| Sodium phosphate ... ... ... | 0.01 |
| Cochineal ... ... ... ... ... | 0.38 |
| Refined sugar ... ... ... ... | 70.00 |
| Orange-flower water ... ... ... | 3.00 |
| Distilled water, sufficient to produce ... | 100.00 |

The above syrup was found to be a very satisfactory preparation, but it is important that the undiluted triple orange-flower water should be used in making it.

The following syrup, which contains a much larger proportion of iron, was also found to yield a satisfactory product, namely:—

SYRUPUS FERRI SUBCHLORIDI, B.P.C.

| | Parts |
|---|---|
| Iron wine ... ... ... ... ... | 3.50 |
| Hydrochloric acid ... ... ... ... | 10.00 |
| Citric acid ... ... ... ... ... | 0.10 |
| Distilled water ... ... ... ... | 6.25 |
| Syrup, sufficient to produce ... ... | 100.00 |

The laxative compound syrup of figs given by the codex, and having the following formula,

SYRUPUS FICORUM COMPOSITUS, B.P.C.

| | Parts. |
|---|---|
| Compound tincture of rhubarb ... ... | 5.00 |
| Liquid extract of senna pods ... ... | 10.00 |
| Spirit of cinnamon ... ... ... | 1.25 |
| Spirit of nutmeg ... ... ... ... | 1.25 |
| Tasteless liquid extract of cascara sagrada | 5.00 |
| Syrup of figs, sufficient to produce ... | 100.00 |

was found to have very little action upon the bowels in most cases, and a much more satisfactory prescription was the following:—

| | Parts. |
|---|---|
| Liquid extract of senna pods ... ... | 3 |
| Liquid extract of liquorice ... ... | 5 |
| Tincture of jalap ... ... ... ... | 5 |
| Syrup of rhubarb ... ... ... ... | 30 |
| Syrup of figs ... ... ... ... | 15 |

Some other useful prescriptions which yielded elegant and stable products, are the following:—

SYRUPUS HYPOPHOSPHITUM COMPOSITUS, B.P.C.

| | Parts. |
|---|---|
| Calcium hypophosphite ... ... ... | 1.00 |
| Manganese hypophosphite ... ... | 0.50 |
| Potassium hypophosphite ... ... | 0.50 |
| Quinine hypophosphite ... ... ... | 0.25 |
| Strychnine ... ... ... ... | 0.012 |
| Refined sugar ... ... ... ... | 70.00 |
| Hypophosphorous acid ... ... ... | 0.625 |
| Strong solution of iron hypophosphite (B.P.C.) ... ... ... ... | 5.00 |
| Chloroform water, sufficient to produce | 100.00 |

The only objection that might be raised to the above syrup, is its colour; but this could merely be on the ground that it is different to that of the usual syrups of hypophosphites.

It is sometimes desired to prescribe formates along with glycerophosphates, and this may very conveniently be done in the form of the Syrupus Glycerophosphatum cum Formitibus, B.P.C., which yields a very satisfactory preparation.

